# Bacterial Diversity Assessment in Antarctic Terrestrial and Aquatic Microbial Mats: A Comparison between Bidirectional Pyrosequencing and Cultivation

**DOI:** 10.1371/journal.pone.0097564

**Published:** 2014-06-02

**Authors:** Bjorn Tytgat, Elie Verleyen, Dagmar Obbels, Karolien Peeters, Aaike De Wever, Sofie D’hondt, Tim De Meyer, Wim Van Criekinge, Wim Vyverman, Anne Willems

**Affiliations:** 1 Laboratory for Microbiology, Department of Biochemistry and Microbiology, Ghent University, Ghent, Belgium; 2 Laboratory of Protistology and Aquatic Ecology, Department of Biology, Ghent University, Ghent, Belgium; 3 Laboratory of Bioinformatics and Computational Genomics, Department of Mathematical Modelling, Statistics and Bioinformatics, Ghent University, Ghent, Belgium; Charité-University Medicine Berlin, Germany

## Abstract

The application of high-throughput sequencing of the 16S rRNA gene has increased the size of microbial diversity datasets by several orders of magnitude, providing improved access to the rare biosphere compared with cultivation-based approaches and more established cultivation-independent techniques. By contrast, cultivation-based approaches allow the retrieval of both common and uncommon bacteria that can grow in the conditions used and provide access to strains for biotechnological applications. We performed bidirectional pyrosequencing of the bacterial 16S rRNA gene diversity in two terrestrial and seven aquatic Antarctic microbial mat samples previously studied by heterotrophic cultivation. While, not unexpectedly, 77.5% of genera recovered by pyrosequencing were not among the isolates, 25.6% of the genera picked up by cultivation were not detected by pyrosequencing. To allow comparison between both techniques, we focused on the five phyla (*Proteobacteria*, *Actinobacteria*, *Bacteroidetes, Firmicutes* and *Deinococcus-Thermus*) recovered by heterotrophic cultivation. Four of these phyla were among the most abundantly recovered by pyrosequencing. Strikingly, there was relatively little overlap between cultivation and the forward and reverse pyrosequencing-based datasets at the genus (17.1–22.2%) and OTU (3.5–3.6%) level (defined on a 97% similarity cut-off level). Comparison of the V1–V2 and V3–V2 datasets of the 16S rRNA gene revealed remarkable differences in number of OTUs and genera recovered. The forward dataset missed 33% of the genera from the reverse dataset despite comprising 50% more OTUs, while the reverse dataset did not contain 40% of the genera of the forward dataset. Similar observations were evident when comparing the forward and reverse cultivation datasets. Our results indicate that the region under consideration can have a large impact on perceived diversity, and should be considered when comparing different datasets. Finally, a high number of OTUs could not be classified using the RDP reference database, suggesting the presence of a large amount of novel diversity.

## Introduction

With its severe physical, chemical, and climatic conditions [1], Antarctica is characterized by harsh environmental settings and hosts communities of well-adapted microbiota that are capable of withstanding selective pressures, such as high UV-radiation, drought, light limitation and extremely low temperatures. These adaptations may therefore be potentially of biotechnological and economical value [2], [3]. Until now, studies have mainly used culturing approaches [4], [5] and a number of culture-independent techniques such as Denaturing Gradient Gel Electrophoresis (DGGE) [6], Terminal Restriction Fragment Length Polymorphism (t-RFLP) [7], [8], Automated Ribosomal Interspacer Analysis (ARISA) [9] and clone libraries [8], [10], [11], [12] to shed light on Antarctic bacterial diversity. These studies reported taxa that are new to science [4], [5], [13] and/or revealed that – as in other regions and environments [14] – Antarctic microbial diversity is much larger than previously thought.

Whereas Next Generation Sequencing (NGS) techniques have now found their way to nearly every environment, ranging from the deep sea [15] to tropical forest soils [16] and the human microbiome [17], the Antarctic region remains relatively underrepresented in these microbial diversity studies. This is surprising, given the fact that the diversity reported with NGS is orders of magnitude higher than that recovered with traditional culturing and Sanger sequencing, and at least one order of magnitude higher than recovered from large clone libraries [18]. More recently NGS has been used to study Antarctic samples, including McMurdo Dry Valley soils [19], [20], soils from Alexander Island [21], rhizosphere bacteria of the only two vascular plants in the Antarctic Peninsula [22], a study of community turnover due to global warming [23], a survey of cyanobacterial diversity in microbial mats [24] and a comparison of seasonal variation in coastal marine bacterioplankton [6]. The relative paucity of Antarctic studies is largely due to the remoteness and vastness of the continent, the harsh environmental conditions and the costs associated with expeditions. Yet, exactly these limitations have kept the environment relatively pristine, thus providing excellent conditions to investigate several questions of particular interest to microbiologists such as to which extent historical processes shape microbial biogeography patterns and the degree of endemism. Moreover, polar regions with their uniquely adapted microbiota are particularly prone to the impact of global warming [25], [26], [27], [28] and microbial diversity data are therefore urgently needed as baseline data for tracking this impact.

Microbial communities typically consist of few high-abundant taxa, with the majority of taxa belonging to the so called rare biosphere [18], [29], [30]. Although it was shown that cultivation is able to pick up some of these rare community members [31], it is generally thought that only through the deep sequencing that NGS offers, this vast diversity can be detected [18], [32]. In turn, this also implies that cultured strains are generally expected to be recovered by pyrosequencing. Here we aimed to test this hypothesis by comparing the diversity of heterotrophic bacterial groups previously recovered from Antarctic microbial mat samples by cultivation with the diversity of the corresponding groups as revealed by 454 pyrosequencing. An additional objective was to assess the impact of the region of the 16S rRNA gene on the diversity data obtained. This was done by comparing forward and reverse pyrosequencing datasets and contrasted with a comparison of forward and reverse data from the cultured strains, where no effects of the pyrosequencing process could be at work.

## Materials and Methods

### Samples Used

Details of the study sites have been described previously [4], [33], [34]. Briefly, two terrestrial and seven limnetic microbial mat samples were collected aseptically during different field campaigns in December/January 2003 (PQ1, TM2 and TM4) and in January 2007 (BB50, BB115, LA3, SK5, WO10 and SO6). One sample (PQ1) was collected on Pourquoi-Pas Island off the west coast of Graham Land (Antarctic Peninsula). All other samples were collected from Eastern Antarctic habitats. The two terrestrial microbial mat samples (BB50 and BB115) were taken near the Utsteinen nunatak in the Sør Rondane Mountains (Dronning Maud Land). Three samples were from Lützow-Holm Bay (Dronning Maud Land), namely from a small saline lake in Langhovde (LA3), from Naka-Tempyo Lake (SK5) in Skarvsnes, and from a small saline pond (WO10) in West Ongul Island. One sample (SO6) was taken from Lake Melkoye (unofficial name) in Schirmacher Oasis (Dronning Maud Land). The two remaining samples were collected in the Transantarctic Mountains. Sample TM2 was taken from Forlidas Pond (Dufek Massif, Pensacola Mountains), while sample TM4 was taken from Lundström Lake (Shackleton Range). All samples were kept frozen during transport and stored at −20°C.

### Processing of 16S rRNA Gene Sequences of Cultures

The cultured heterotrophic bacterial diversity of these samples was reported earlier [4], [33], [34], [35]. From these, we selected 1,666 high quality sequences for comparison with pyrosequencing. To allow this comparison, the sequences from bacterial cultures were aligned to the Silva reference database [36], and trimmed so as to cover the alignment of the sequences obtained using pyrosequencing (see below). They were further processed together with both forward and reverse pyrosequencing datasets.

### Pyrosequencing

To allow direct comparison, DNA was extracted from the same frozen samples previously used for the cultivation experiments using 5 g per sample. Extracellular DNA was first removed as described by Corinaldesi *et al*. [37] and DNA extraction was subsequently performed according to Zwart *et al*. [38]. Sequencing of the 16S rRNA V1–V3 regions was performed using forward primer pA (AGAGTTTGATCCTGGCTCAG 8–27) [39] and reverse primer BKL1 (GTATTACCGCGGCTGCTGGCA 536–516). Because it proved impossible to concatenate the complementary reads due to insufficient overlap, the forward and reverse sequences were analyzed separately. The forward reads hence cover the complete V1 and V2 regions, whereas the reverse reads cover the V3 and part of the V2 region for the longest sequences [40].

Multiplexing was done with barcodes proposed by Parameswaran *et al*. [41]. Each PCR mixture contained 1–2 µl of template DNA, 2 µl of fusion primers and barcodes (10 µM), 2.5 µl dNTPs (10 mM), 1.5 µl of 10x buffer, 0.25 µl of 5 U/µl FastStart High Fidelity Polymerase (Roche) and was adjusted to a final volume of 25 µl with sterile HPLC-water. PCR cycling included 3 min at 94°C, followed by 35 cycles of 94°C for 30 s, 55°C for 60 s and 72°C for 90 s and finally 8 min at 72°C. PCR products were purified using a High Pure PCR Product Purification Kit (Roche). Finally, pyrosequencing was performed on a Roche 454 GS FLX Titanium machine at NXTGNT (Ghent, Belgium) after quality control of the DNA with a Qubit 2.0 Fluorometer (Life Technologies) and a Bioanalyzer (Agilent Technologies).

Raw sequences are available from the NCBI Sequence Read Archive under accession numbers SRR1146576 and SRR1146579.

### Processing of Pyrosequences

The obtained reads were processed using Mothur [42] version 1.27.0, generally following Schloss *et al*. [43] and the Mothur SOP (http://www.mothur.org/wiki/454_SOP; version of 6 November 2012). The data were denoized using Mothur’s PyroNoise [44] implementation with 450 flows as the minimal flow length and trimming of the longer sequences to this length [43]. Overall, the minimal required sequence length was set at 200 nucleotides (nt). To avoid poor sequence quality, no ambiguous bases (N) were allowed [45] and sequences with homopolymers longer than 8 nt were culled, as it is known that long homopolymers are problematic for 454 pyrosequencing [43], [46], [47]. The sequences were aligned using Mothur’s alignment command, based on the GreenGenes NAST aligner [48] with default parameters and the Silva reference database [36], which takes into account the secondary structure of the 16S SSU rRNA. The starting and ending positions of the alignment were checked to ensure that sequences were overlapping the same alignment space. Sequences not starting at the correct position or ending before 95% of all the sequences were removed from the analysis. To increase computational speed and decrease data size, duplicate (identical) sequences were temporarily removed using the unique.seqs command. Further correction for erroneous base calls was done using single linkage preclustering according to Huse *et al*. [49]. Next, we used Uchime [50] with default parameters for intra-sample *de novo* chimera checking. Positively identified chimeric sequences were removed from further analyses.

### Sequence Identification and OTU Clustering

Sequences were identified using Mothur’s implementation of the RDP classifier [51] by means of the modified RDP training-set release 9 (available at http://www.mothur.org/wiki/RDP_reference_files) at an 80% bootstrap value. The RDP database was chosen so that a comparison with the original cultivation data was possible, despite its known limitations because of its small size [52], [53], possibly overestimating the number of unclassified OTUs. This training set too was first aligned and trimmed to the alignment space of the query sequences, increasing confidence values and reducing the number of unclassified sequences [52]. Non-cyanobacterial “chloroplast” sequences were removed from the dataset. Distances were calculated (dist.seqs command, default settings), after which the sequences were clustered using the average neighbor joining algorithm to generate OTUs at a 97% cutoff level [54].

### SIMPROF Analysis

In order to compare the community composition obtained using culturing versus pyrosequencing a SIMPROF analysis [55] was performed using Primer 6 [56]. SIMPROF is a permutation-based procedure that ranks the pairwise similarities in each group and tests the null hypothesis that samples were all drawn from the same species assemblage. Because the number of sequences is consistently higher in the pyrosequencing dataset, we standardized the number of sequences in each sample to the lowest number of sequences obtained in all of the samples (i.e. 119 forward and 116 reverse sequences in sample LA3). To achieve this, we randomly sampled this number of sequences from each sample with replacement. This procedure was done 5 times, which resulted in 5 subsets for each sample. First, a Jaccard similarity matrix was constructed and subsequently used to undertake a group-average cluster analysis. Second, to ascertain the level of structure present in the groups formed by each dendrogram, a SIMPROF test with 10,000 simulations and the stopping rule specified at the 5% significance level was run. This was done for both forward and reverse datasets.

## Results

### Sequence Data of Bacterial Isolates

**Figure 1:**
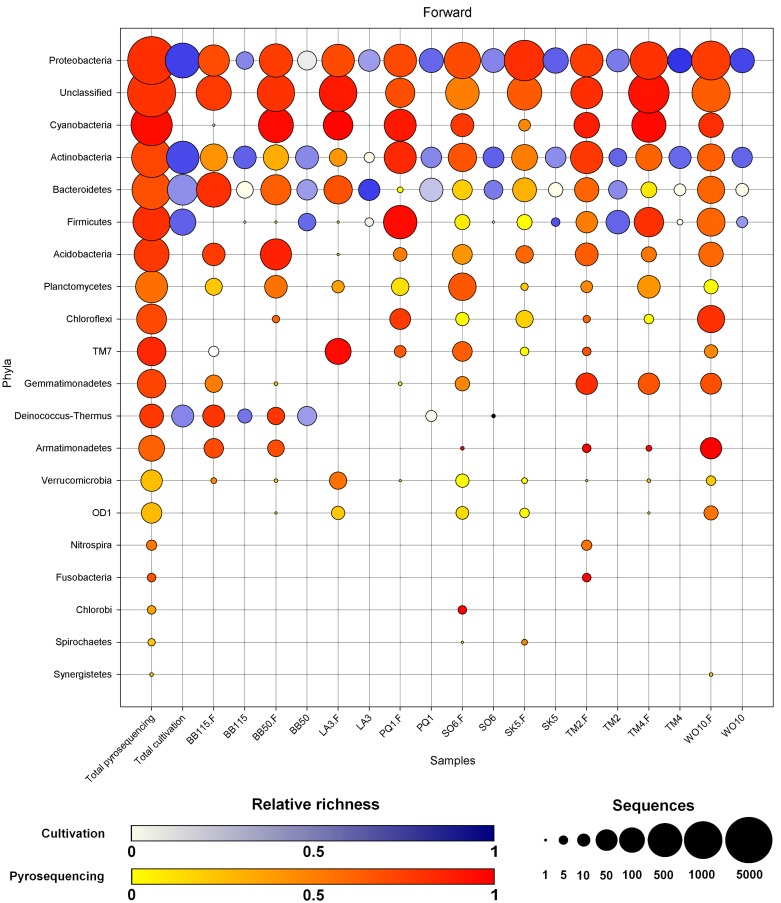
Overview of the distribution of the phyla per sample for the forward sequencing dataset. Circle area is a log_2_ transformation of the number of sequences ([log_2_(N)*5/PI], with N the number of sequences in that phylum). Color intensity reflects the number of OTUs per phylum (total OTUs/total sequences), with a darker hue indicating a higher relative richness. The first two columns show the total number of sequences and diversity of each phylum for pyrosequencing and cultivation separately. The phyla are ordered according to decreasing total number of sequences. The yellow to red scale shows pyrosequencing data, the blue-purple scale the cultivation data.

Of the initial 1,666 sequences, 1,578 remained after the forward processing together with the pyrosequences. This was mainly due to the removal of sequences that did not match the correct starting or ending positions of the alignment space. A total of 342 OTUs in 76 genera from five different phyla were obtained (Figure 1). Most of the OTUs belonged to the phyla *Bacteroidetes* and *Proteobacteria*, with 107 and 106 members respectively. *Actinobacteria* was the third best represented phylum with 78 OTUs, followed by *Firmicutes* and *Deinococcus-Thermus* with 31 and 20 OTUs respectively.

**Figure 2:**
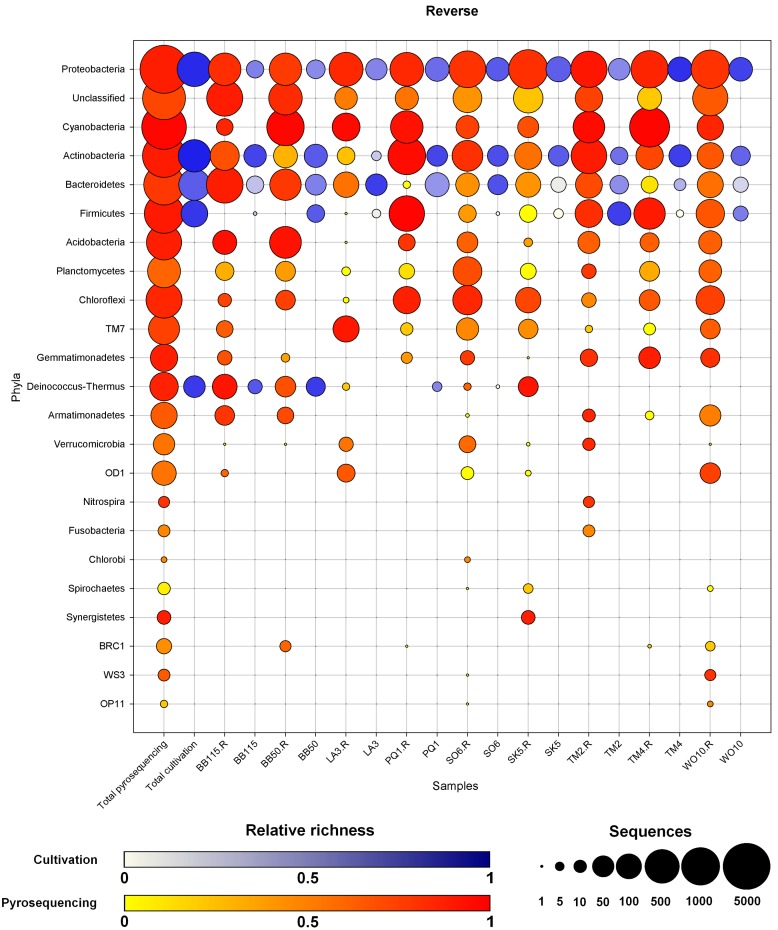
Overview of the distribution of the phyla per sample for the reverse sequencing dataset. Circle area is a log_2_ transformation of the number of sequences ([log_2_(N)*5/PI], with N the number of sequences in that phylum). Color intensity is an approximation for the number of OTUs per sequence (total OTUs/total sequences). The first two columns show the total number of sequences and diversity of each phylum for pyrosequencing and cultivation separately. The order of the phyla is as in Figure 1 and additional phyla were added at the bottom. The yellow to red scale shows pyrosequencing data, the blue-purple scale the cultivation data.

The initial 1,666 sequences were also subjected to the reverse processing pipeline. In contrast to the 1,578 forward sequences, this yielded only 1,519 sequences divided over 214 OTUs in 61 genera. The relative proportion of the phyla did not differ drastically when processed through the forward or reverse pipeline (Figures 1 and 2), although only 51 genera were shared between the forward and reverse dataset of the isolates. In total, we identified 86 genera for the combined processed cultivation results, while some sequences remained unclassified. Of these 86 genera, 20 (23% of cultivated genera) were not picked up by pyrosequencing.

Heatmaps showing the distribution of the most frequently recovered OTUs based on the forward (Figure S1) and reverse (Figure S2) cultivation sequences, revealed that many of these OTUs were shared between samples.

### Pyrosequencing Data

#### Forward dataset

After processing the forward pyrosequencing data, 23,510 high quality sequences were left (on average 2,612±829 per sample); they were on average 243±14 nt long. The chimera content per sample in the forward dataset ranged from 0.1% (TM2) to 5.8% (SK5) of sequences (Table S1). For eight samples, in the non-redundant dataset (i.e. dataset filtered for duplicate sequences), the percentage of chimeras was higher than when considering the complete dataset, indicating that many chimeras were singletons or low-abundant sequences.

We observed 2,940 OTUs of which 947 remained unclassified at the phylum level (represented by 7,659 sequences) and 2,066 (15,271 sequences) at the genus level. Per sample, the number of OTUs unclassified at the phylum level varied between 40 (TM4) and 274 (WO10). The identified OTUs belonged to 220 genera in 19 phyla (Tables S2 and S3 respectively). *Proteobacteria*, *Cyanobacteria*, *Actinobacteria*, *Bacteroidetes*, *Firmicutes*, *Acidobacteria* and *Planctomycetes* were present in every sample (Figure 1), although relative number and OTU richness could differ drastically. Cyanobacteria were well represented in most samples, but less so in SK5 and BB115. *Deinococcus-Thermus* was relatively well recovered and showed a high richness in the terrestrial samples (BB50 and BB115).

**Figure 3:**
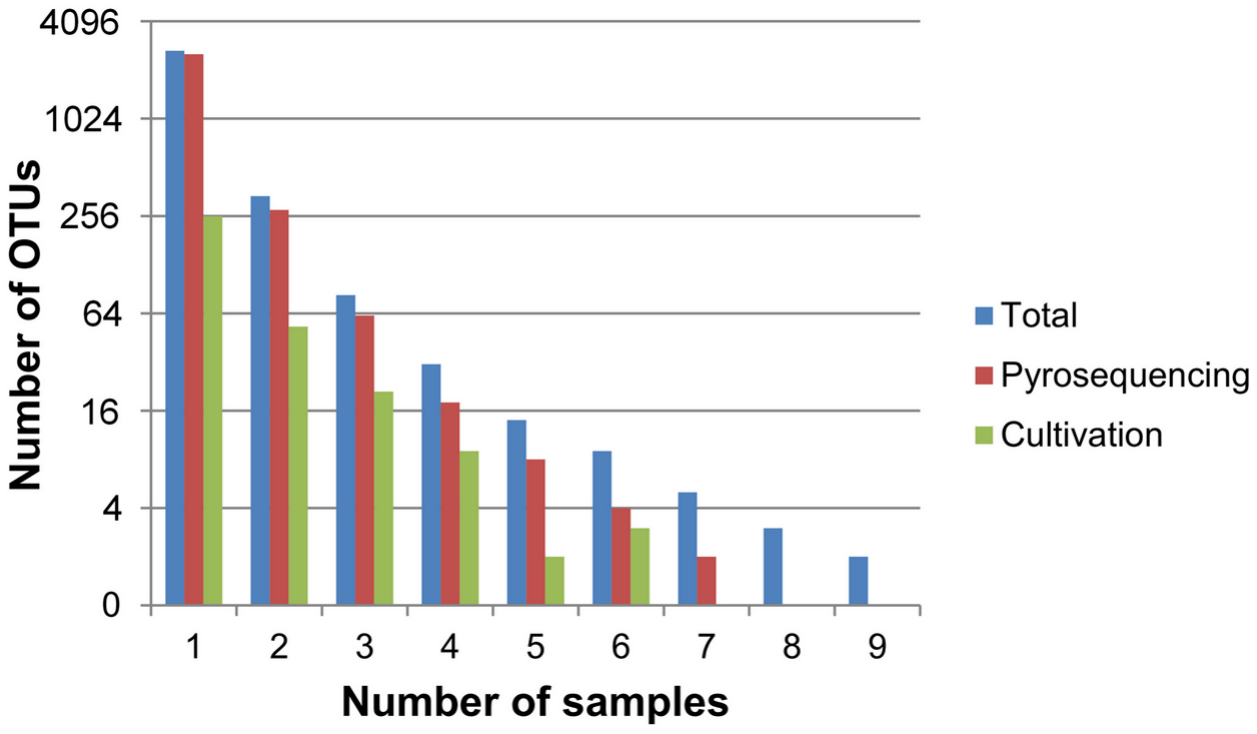
Bar chart illustrating the number of OTUs picked up from one or more samples for the forward dataset. The number of OTUs is log_2_ transformed. Blue bars, total sequences (pyrosequences plus cultivated sequences); red bars, pyrosequences only; green bars, cultivation sequences only.

A total of 2,693 (84.9%) of the OTUs were restricted to one sample (Figure 3), and 1,464 (46.2%) were effectively singletons (i.e. represented by only one sequence).

The most abundant OTU (OTU3056) was represented by 2,216 sequences, nearly three times as many as the second most abundant OTU (OTU0858, 871 sequences), and was found in six out of the nine samples (BB115, BB50, PQ1, TM2, TM4 and WO10). It was not picked up through cultivation and was not identified using our RDP training set. A separate blast against the GreenGenes database [57], however, revealed that it was identical to *Phormidium autumnale* str. Arct-Ph5 (*Cyanobacteria*, a group not targeted by the cultivation experiments). None of the OTUs was found in every sample through pyrosequencing in the forward dataset. One OTU (OTU2885; *Rhizobacter*, *Gammaproteobacteria*) was found in seven samples. Five OTUs were recovered by pyrosequencing from six samples, including the aforementioned cyanobacterial OTU3056, a *Polaromonas* (OTU2491, which was also cultured) and a *Herbaspirillum* species (both *Betaproteobacteria*), and a *Methylobacterium* species (*Alphaproteobacteria*) and finally OTU2399, identified as *Brevundimonas* sp. (*Alphaproteobacteria*), which was actually retrieved from every sample (i.e. it was recovered through either cultivation, pyrosequencing or in some samples by both). All other OTUs were only recovered from five samples or less.

The combined number of OTUs from forward pyrosequencing and cultivation was 3,172 (totaling 25,088 sequences). Only 110 OTUs were shared between both approaches and 232 were restricted to the cultivation data. A heatmap (Figure S3) showing the distribution of the most frequently recovered pyrotag OTUs, revealed that few of these OTUs were shared between samples. In fact, most of these high-abundant OTUs were merely recovered from one or two samples. The SIMPROF analysis revealed that the community structure in all samples assessed using pyrosequencing is significantly different from that analyzed using culturing (Figure S4). Not unexpectedly, given cultivation bias, the similarity between samples analyzed with the culturing approach is higher. However, these observations were consistent when taking into account only the five phyla that were recovered by both approaches (data not shown).

#### Reverse dataset

Reverse pyrosequencing starting from the end of the V3 region resulted in 22,778 high quality sequences after processing. The chimera content was generally higher than for the forward pyrotags for all samples (Table S1). Particularly in sample SK5, up to 43.4% of the non-redundant sequences were identified as chimeras by Uchime, resulting in the removal of 23% of all sequences in that sample. Also for sample PQ1 23% of all sequences were removed, while only 19.6% of the unique sequences were flagged as chimeras, indicating a substantial proportion of chimeras in this sample. We obtained only 1,983 OTUs overall, of which 485 remained unclassified at phylum level (2,776 sequences) while the rest belonged to 22 phyla (Figure 2, Table S3). We were able to identify 197 genera in the reverse dataset (Table S2). The taxonomy at genus level remained unresolved for 1,376 OTUs (12,295 sequences). Although considerably fewer OTUs were observed in the reverse dataset, the distribution over phyla were similar to these observed for the forward pyrosequences (Figure 2, Table S3). The number of sequences unclassified at phylum level (485 OTUs, 2,776 sequences) was much smaller than in the forward sequencing (947 OTUs, 7,659 sequences) and represented 24% versus 32% of the OTUs, respectively. Compared to the forward dataset, *Deinococcus-Thermus* was additionally picked up from samples LA3, SO6 and especially SK5 (Figure 2). Also *Cyanobacteria* and *Chloroflexi* were generally more abundantly picked up by the reverse sequencing, and additionally, three extra bacterial phyla were recovered: WS3, OP11 and BRC1. Phylum BRC1 was obtained from four different samples (BB50, PQ1, TM4 and WO10) with six OTUs in total; WRC3 was represented by two OTUs, one from SO6 and a second one from WO10; OP11 was also found in these two latter samples. The number of singleton OTUs was lower for the reverse dataset: 476 (24%) here vs. 897 (31%) in the forward dataset. This discrepancy equals 44% of the difference in the total number of OTUs obtained between both datasets (1,983 in the reverse dataset compared to 2,940).

Heatmaps showing the distribution of the OTUs most abundantly recovered in the reverse pyrosequencing data (Figure S5) and in the reverse cultivation dataset (Figure S2) reveal generally similar trends as for the forward sequencing (Figures S1 and S3). However, nine OTUs (1942, 1956, 1959, 2036, 2043, 2044, 2064, 2109 and 2115) in the high-abundant reverse pyrosequencing selection were also found in the cultivated dataset, which is considerably more than for the forward dataset. Especially OTU2109 (*Sphingomonadaceae* sp., *Alphaproteobacteria*) was recovered well through cultivation (not found in sample TM4), and pyrosequencing (not found in sample BB115). OTU1849 (*Methylobacterium*, *Alphaproteobacteria*) was recovered from all pyrosequencing samples. Four unclassified OTUs were recovered from eight samples (three alphaproteobacteria and one actinobacterium). The most abundant OTU (OTU1804) with 1,226 sequences was found in five samples. It was classified as an unknown cyanobacterial order by the RDP training set. Again, a blast against the Greengenes database resulted in *P*. *autumnale* (strains Ant-Ph68 and Arct-Ph5, both with an identity score of 100). Similar to the forward dataset, both techniques resulted in significantly different clusters and the variability between the different samples is higher in the datasets obtained through pyrosequencing (Figure S6).

## Discussion

### Comparison of Forward and Reverse Datasets

Two terrestrial and seven aquatic Antarctic microbial mat samples were subjected to bidirectional pyrosequencing of the V1 to V3 variable region of the 16S rRNA gene. After processing, the forward dataset spanned the V1 and V2 variable regions, while the reverse dataset covered the V3 and part of the V2 variable regions. The comparison of bidirectional sequencing revealed large differences in the number of OTUs recovered, although the number of sequences and genera was generally comparable. More in particular, the number of OTUs was about 50% higher for the forward dataset compared to the reverse dataset. This is in part likely due to the V1 region being more variable than the more conserved V3 region [58], [59], [60]. Hence, the traditionally used cut-off values (e.g. 95% as a proxy for genus level, or 97% for species level) which have proven to be insufficient or inadequate for all taxa [61], might additionally require modification for different regions of the 16S rRNA gene. Highly variable regions such as V1 could be clustered using lower values (for example 97%) than more conserved regions (e.g. V3 or V6), which might require a higher (e.g. 99%) identity cut-off. These considerations should be taken into account when selecting the region to analyze, but also when comparing studies and diversity data based on different variable regions [62]. Not only did the number of OTUs differ between both regions, identification was affected too. For example, although the number of genera identified from the forward and the reverse dataset was broadly similar (220 vs. 197), only 132 or 67% of the genera identified from the reverse dataset were also present in the forward dataset, corresponding to 60% of the genera in the forward dataset. The combined number of genera based on the RDP training set was 285. Similarly, for the Sanger sequences of the cultures, comparison of forward and reverse trimmed dataset revealed 76 and 61 genera respectively, of which 51 were in common. As pyrosequencing artifacts cannot have been introduced in the cultivation dataset, these differences highlight the impact of the variable zones covered on the outcome of the genus identifications. With the continuous development of NGS techniques, the significance of this problem can be expected to reduce with increasing read length.

Another striking difference between the sequencing directions was that the number of chimeras was higher in the reverse dataset (Table S1). This is probably also due to the differences in variability of the regions targeted; the more conserved V3 region might be more likely to function as a template for annealing than V1, especially between closely related taxa [63], [64]. Furthermore, not only do PCR conditions (such as extension times and the number of PCR cycles) or conserved regions affect chimera formation [43], [64], [65], it has been shown that certain positions in the 16S rRNA gene are more prone to chimera formation [63]. This implies that chimeras are not necessarily restricted to low-abundant sequences, questioning the removal of only OTUs with a low abundance, a common practice to reduce artifacts.

### Contrast between Diversity Data from Pyrosequencing and Cultivation

The comparison of the bacterial diversity estimate obtained by bidirectional 454 pyrosequencing with the results from previous cultivation studies [4], [33], [34] unsurprisingly confirmed that pyrosequencing results in a higher diversity (in total 22 phyla, 285 genera) than obtained through culturing (5 phyla, 86 genera). Indeed, we observed a striking and significant difference in taxonomic composition and abundance of groups recovered using both methods, with communities standardized to the lowest number of sequences (Figures S4 and S6). This likely results from the obvious bias related to the specific cultivation conditions used, which were set to target mostly heterotrophic, aerobic and psychrophilic or psychrotolerant bacteria [4]. Some of the phyla that were detected by pyrosequencing but not picked up through cultivation included groups that were not targeted such as anaerobes (e.g. *Clostridium* which was frequently recovered in sample PQ1), phototrophic *Cyanobacteria* and *Chloroflexi*, or groups for which cultivation is not yet optimized and that have very few or even no cultured representatives (e.g. *Acidobacteria*, *Planctomycetes*, *Verrucomicrobia*, *Armatimonadetes*, TM7; see Table S3). Given that only heterotrophic bacteria had been targeted in the isolation campaigns and a limited set of cultivation conditions was tested, a comparison with pyrosequencing is only possible to a very limited extent. We tried to take this into account by further focusing this part of the discussion on the OTUs and named genera of the five phyla picked up by both techniques (*Actinobacteria*, *Bacteroidetes*, *Deinococcus-Thermus*, *Firmicutes* and *Proteobacteria*). This restricted comparison confirmed the general observation that pyrosequencing can detect more diversity at all taxonomic levels. Nevertheless, particularly at lower taxonomic and phylogenetic levels (OTUs, genera), we found extremely little overlap in the diversity between both datasets. For example, in the forward sequencing datasets, of the 342 OTUs recovered using culturing, 232 (67.8%) were not picked-up by pyrosequencing. For these five phyla, a total of 204 genera were identified, of which 51 were in common, 131 were unique for pyrosequencing and 22 unique for cultivation. Thus about 30% of cultured genera were not detected in our pyrosequencing data (e.g. the *Firmicutes* genus *Paenibacillus*; see Table S2). Reverse sequencing showed generally analogous results.

In addition to the above mentioned cultivation bias, at least three other non-mutually exclusive processes might underlie the significant differences between the cultivation and pyrosequencing datasets. Firstly, manual picking of individual colonies for further characterization in culture-based approaches introduces an additional bias. The sheer quantity of isolates makes it nearly impossible to select and cultivate every colony separately, especially when the number of samples is high. Phenotypic (morphological) selection may thus lead to an underestimation of the genotypic diversity, because macroscopically identical colonies might in fact represent different OTUs, whether closely related or not. Secondly, the failure of pyrosequencing to detect the majority of the cultured organisms could indicate that our sequencing depth was not large enough (Figures S7 and S8), which is often the case for large scale surveys [66], or that low-abundant organisms were missed because they were below the detection limit of the technique [67]. Thirdly, while sequencing depth is one aspect, PCR-related biases (e.g. GC-content) and sequencing errors (e.g. homopolymers) may also contribute to the observed differences [68], [69], [70]. A GC-content deviating strongly from 50% may induce a PCR-bias and this could explain why certain OTUs were not detected through pyrosequencing. However, calculation of the %GC of the cultivation-only sequences, in combination with the high number of such OTUs (67.8% of the cultivation OTUs), dismissed this hypothesis in our case (Table 1). Although our preprocessing was done rigorously, e.g. [43], [45], [50], we cannot exclude the possibility that some erroneous sequences have slipped through [32]. Nevertheless, the limited overlap between culturing and pyrosequencing data is in line with observations from comparisons of cultivation and other culture-independent techniques (e.g. clone libraries) in other ecosystems [71]. High-throughput culturing [72] and the use of more diverse growth conditions [73], [74] would probably show that the actual overlap is (much) larger than our results currently suggest. Indeed, extending the incubation time (e.g. up to three months) might reveal additional rare community members [75]. Moreover, cultivation is even able to detect novel organisms where culture-independent techniques fail [74]. It has been proposed that 5000 denoised reads may be needed to describe 90% of the alpha-diversity of 15–20.000 reads and that because of the huge bacterial diversity, almost an infinite number of individuals might need to be identified to accurately describe communities [76].

**Table 1:** Comparison of the GC content of the cultivation-only sequences with the overall values.

	Forward sequencing (V1–V2 regions)			Reverse sequencing (V3–V2 regions)		
	Average (%)	Minimum (%)	Maximum (%)	Average (%)	Minimum (%)	Maximum (%)
All sequences	54	28	70	56	34	67
Cultivation-only^a^	52	43	63	55	51	66

a Sequences from cultivation that were not picked up by pyrosequencing.

**Figure 4:**
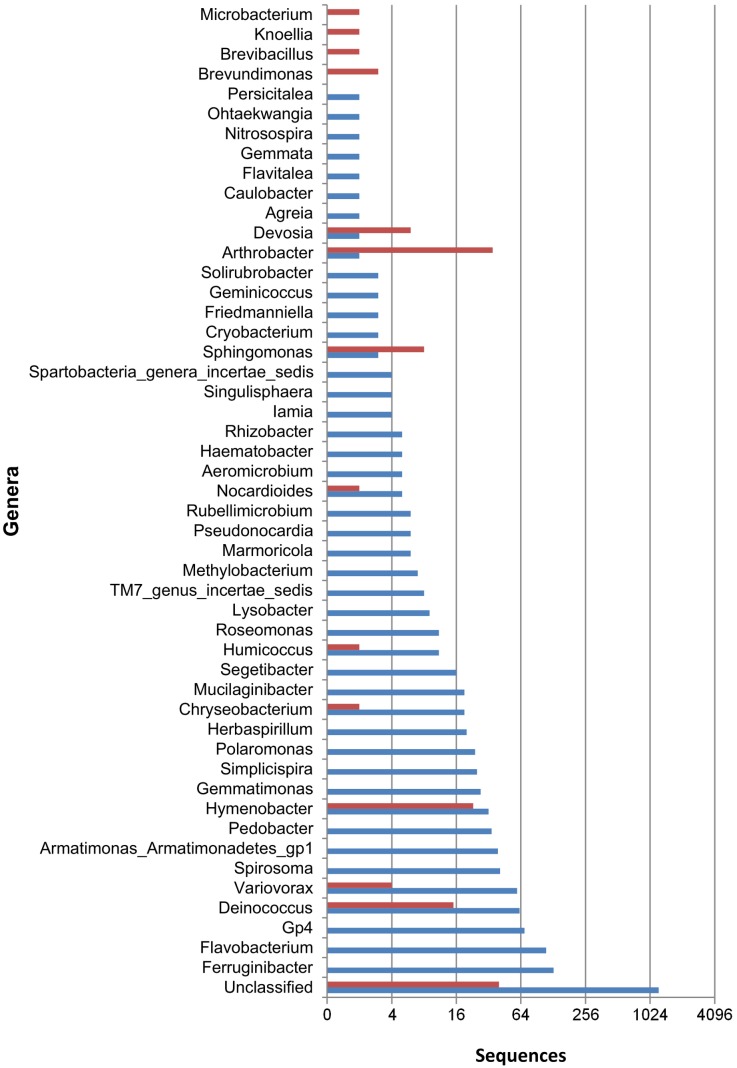
Rank-abundance plot showing the distribution of genera in a sample, illustrating the difference between techniques. Sequence numbers are plotted on a log scale. Blue bars are pyrosequencing based, red bars are cultivation based.

Our comparison further confirmed that even low-abundant but widely distributed organisms can be picked up by both techniques. As an example, Figure 4 shows the distribution of genera in sample BB115 where, typically, the majority of genera are represented by only one or two sequences, some are moderately abundant and a few are very abundant taxa. That cultivation can pick up low-abundant bacteria may often be the result of cultivation conditions allowing enrichment of these taxa. For example, OTU 2399 (*Brevundimonas* sp., *Alphaproteobacteria*) was recovered from sample SK5 six times through pyrosequencing, while 38 times through cultivation. The ability of cultivation to pick up organisms from the rare biosphere was also demonstrated by Shade *et al*. [31], and these and our results show that the nutritional or cultivation requirements of these rare organisms are not necessarily extensive [72]. In fact, *Escherichia coli* is probably the best example to demonstrate this fact. While readily cultured and even functioning as a Gram-negative model organism, it is not a very abundant organism in the human gut [77].

Of the high-abundant OTUs (i.e. having more than 80 sequences) obtained by forward pyrosequencing, only three were also retrieved through cultivation (Figure S3). OTU2742 (*Porphyrobacter*, *Alphaproteobacteria*) was detected through pyrosequencing in samples LA3, PQ1, TM4, SO6 and WO10, and recovered by cultivation from SK5 and PQ1. A second OTU (OTU1961; unclassified alphaproteobacterium) found in BB115, BB50 and PQ1 was also found in two culture samples (SK5 and BB115). Strikingly, neither of these was found in the pyrosequence data of sample SK5. Finally, OTU2229 (*Sphingopyxis*, *Alphaproteobacteria*) was recovered from samples TM2 and WO10 through cultivation, and from samples SK5, SO6 and LA3 by pyrosequencing. In contrast, most of the OTUs frequently obtained via culturing (more than 10 sequences) were also picked up from the same sample by pyrosequencing, although generally at a lower relative abundance than through cultivation (Figure S1). Moreover, no OTU was shared and present at a high relative abundance in both datasets. In the reverse pyrosequencing dataset nine of the frequently recovered OTUs were also picked up by cultivation. One of these (OTU2043, unclassified alphaproteobacterium) was among the high-abundant OTUs in both techniques (Figures S2 and S5).

### Notable Diversity Observations

While *Cyanobacteria* was the dominant phylum of photosynthetic bacteria in all samples, also the phylum *Chloroflexi* was present in all samples. Remarkably, diversity was considerably less in the forward dataset (47 OTUs including genera *Leptolinea* and *Chloroflexus*) than in the reverse dataset (75 OTUs including *Leptolinea*, *Levilinea*, *Caldilinea*, *Heliothrix*, *Herpetosiphon*, *Dehalogenimonas*, *Sphaerobacter*). The genus *Caldilinea*, originally described for thermophilic filamentous bacteria [78], [79], was present in all samples (Table S2). The phylum *Chlorobi* was much less well represented (2 OTUs in one sample).

The phylum *Planctomycetes* was also well represented in all samples: eight genera were detected, although the diversity differed between samples. Notable is the relatively frequent presence of the unusual freshwater genus *Gemmata* [80] with 29 OTUs found in seven of the nine samples in the forward dataset (Table S2).

The genus *Deinococcus* was frequently recovered in the terrestrial samples, which was also especially obvious through cultivation (BB50). Among limnetic mat samples, this genus was only recovered from PQ1 and SO6 by cultivation and was not picked up by pyrosequencing (Table S2). The more exposed nature of terrestrial sites may provide habitats that are particularly suited to *Deinococcus* species which are known for their resistance to radiation and desiccation [81].

A small number of genera were relatively frequently detected in the pyrosequencing data of both terrestrial samples (BB50 and BB115) but rarely in the seven aquatic samples: *Hymenobacter* (30 OTUs terrestrial vs. 5 OTUs in one aquatic sample), *Spirosoma* (17 OTUs terrestrial vs. 1 OTU in one aquatic sample) and *Deinococcus* (12 OTUs terrestrial samples only). Conversely, a considerable diversity of the aquatic and clinical genus *Legionella* was picked up from the aquatic mat samples (62 OTUs in the forward and 39 in the reverse dataset from 6 or 5 of the samples) while no *Legionella* was detected in the terrestrial mat samples.

Pyrosequencing allowed us to obtain a considerable number of OTUs which are as yet unidentified at the genus level (e.g. 70.27% in the forward dataset) in addition to the potentially new taxa already detected through cultivation [4], [33], [34]. These might represent novel diversity adapted to the pristine and unique environment of Antarctica. This high number of novel sequences is comparable to other NGS studies in extreme and as yet understudied habitats. For example, 46% of the sequences from an acidic Andean hot spring remained unclassified at the phylum level [82]. However, the high number of novel sequences might in part also be related to (i) the database used (RDP) which contains a relatively low number of sequences, but is of high quality, and (ii) the presence of artifacts that could inflate the diversity. Indeed, in view of the many possible factors that can increase the sequence diversity, pyrosequencing data are often extensively filtered to remove flawed and chimeric sequences [43], [44], [45], [49], [50], [83]. The sequence processing pipeline used here might reduce the error rate down to 0.02% [43]. We therefore assumed that the remaining sequences are of considerable quality, and that most remaining sequencing errors would be masked by clustering. Clustering of the OTUs at 95% similarity did not result in a large reduction of the number of OTUs or singletons (data not shown), indicating considerable diversity among the OTUs left. Our chimera filtering removed 2.5% and 5.7% of the total sequences in the forward and reverse data respectively. We opted not to remove the singletons and low-abundant sequences because (i) our approach already eliminated 16.6% (forward data) and 43.4% (reverse data) of the non-redundant sequences, and (ii) removing singletons may eradicate not only low quality sequences, but also biologically relevant sequences and novel taxa. In fact, 26 out of the 110 OTUs (23.6%) shared by both pyrosequencing and cultivation were singletons in the forward pyrosequencing data that were thus readily picked up from one or more samples through cultivation. In the reverse dataset the singletons comprised 8 of the 77 overlapping OTUs (10%). These high levels indicate that indiscriminate removal of all singletons would eliminate a considerable portion of the actual diversity.

## Conclusions

Next Generation Sequencing techniques such as 454 pyrosequencing allow a much deeper sampling of microbial communities compared to the more traditional techniques. Our study revealed many unidentified OTUs and showed that the terrestrial and lacustrine bacterial diversity in Antarctica is orders of magnitude larger than previously believed. The comparison between NGS and culturing revealed that both techniques are complimentary and that only a limited number of OTUs is shared between both datasets. Although only a small number of these organisms were cultured, cultivation was able to pick up organisms from the rare biosphere, including organisms that were not recovered from pyrosequencing. With more sequencing depth and increasing read length, this may improve. It is clear that despite the ongoing technological developments, cultivation remains a useful method to uncover unknown diversity, and is currently certainly still needed for the physiological characterization and unambiguous identification of these organisms. Our comparison of forward (covering V1 and V2) and reverse sequences (covering V3 and part of V2) also revealed considerable differences in diversity obtained between variable regions and differences in the number of chimeras present. These aspects should be considered when comparing different studies.

## Supporting Information

Figure S1 Heatmap showing the distribution of the most abundant OTUs based on the forward cultivation sequences**.** These high abundant OTUs are represented by at least 10 sequences. Pyrosequenced samples have the suffix.*F*.
(TIFF)

Figure S2 Heatmap showing the distribution of the most abundant OTUs based on the reverse cultivation sequences. These high abundant OTUs are represented by at least 10 sequences. Pyrosequenced samples have the suffix.*R*.
(TIFF)

Figure S3 Heatmap showing the distribution of the most abundant OTUs based on forward pyrosequencing. These high abundant OTUs are represented by at least 80 sequences. Pyrosequenced samples have the suffix.*F*.
(TIFF)

Figure S4 SIMPROF showing the clustering of the forward dataset. Each sample was subsampled 5 times with replacement to the lowest number of sequences (119 in cultured sample LA3). Full (black) lines are significant, dashed (red) lines are not.
(TIF)

Figure S5 Heatmap showing the distribution of the most abundant OTUs based on reverse pyrosequencing. These high abundant OTUs are represented by at least 100 sequences. Pyrosequenced samples have the suffix.*R*.
(TIFF)

Figure S6 SIMPROF showing the clustering of the reverse dataset. Each sample was subsampled 5 times with replacement to the lowest number of sequences (116 in cultured sample LA3). Full (black) lines are significant, dashed (red) lines are not.
(TIF)

Figure S7 Rarefaction of the forward sequenced samples.
(TIF)

Figure S8 Rarefaction of the reverse sequenced samples.
(TIF)

Table S1 Per sample chimera content for both sequencing directions.
(XLSX)

Table S2 Overview of the genera recovered. The number of OTUs within each genus is shown per sample for both pyrosequencing and cultivation.
(XLSX)

Table S3 Summary of the number of sequences and OTUs at the phylum level.
(XLSX)
