# Supplementary figures and images for: Bacterial Diversity Assessment in Antarctic Terrestrial and Aquatic Microbial Mats: A Comparison between Bidirectional Pyrosequencing and Cultivation
Source: PLoS One. 2014 Jun 2;9(6):e97564. doi: 10.1371/journal.pone.0097564 (PMC4041716; doi:10.1371/journal.pone.0097564)

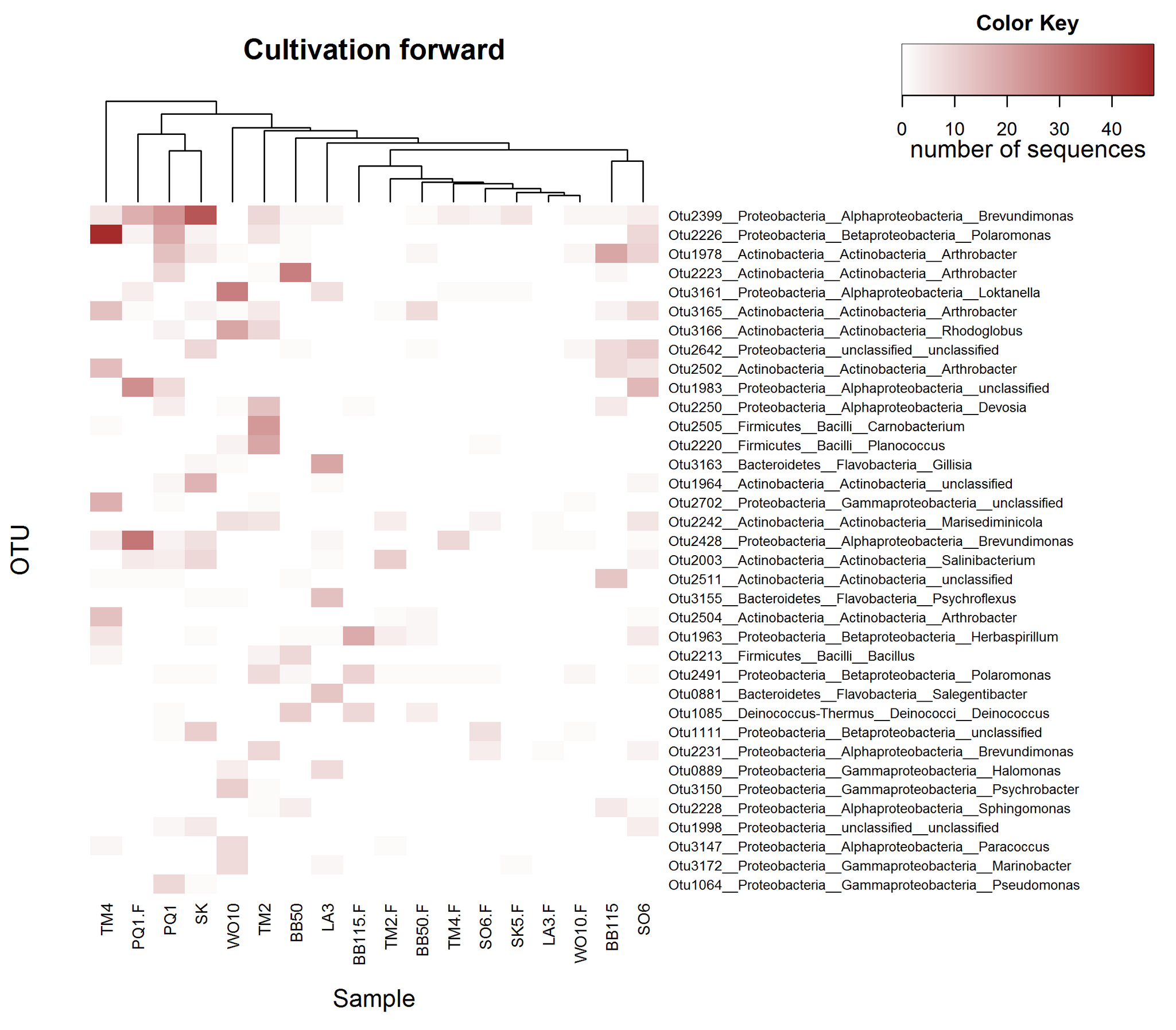

Supplement: Figure S1 — Heatmap showing the distribution of the most abundant OTUs based on the forward cultivation sequences. These high abundant OTUs are represented by at least 10 sequences. Pyrosequenced samples have the suffix.F. (TIFF) [file pone.0097564.s001.tiff]

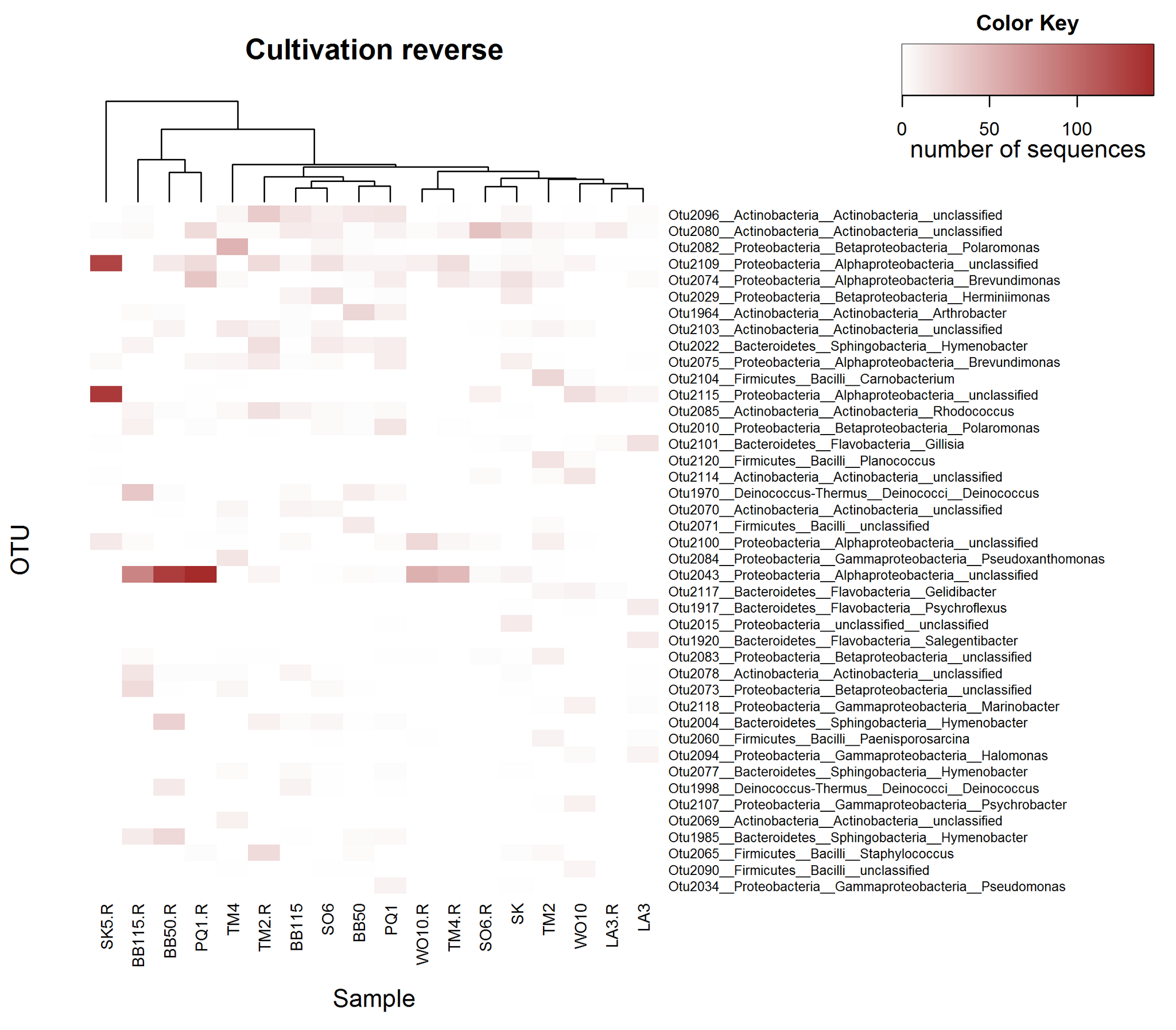

Supplement: Figure S2 — Heatmap showing the distribution of the most abundant OTUs based on the reverse cultivation sequences. These high abundant OTUs are represented by at least 10 sequences. Pyrosequenced samples have the suffix.R. (TIFF) [file pone.0097564.s002.tiff]

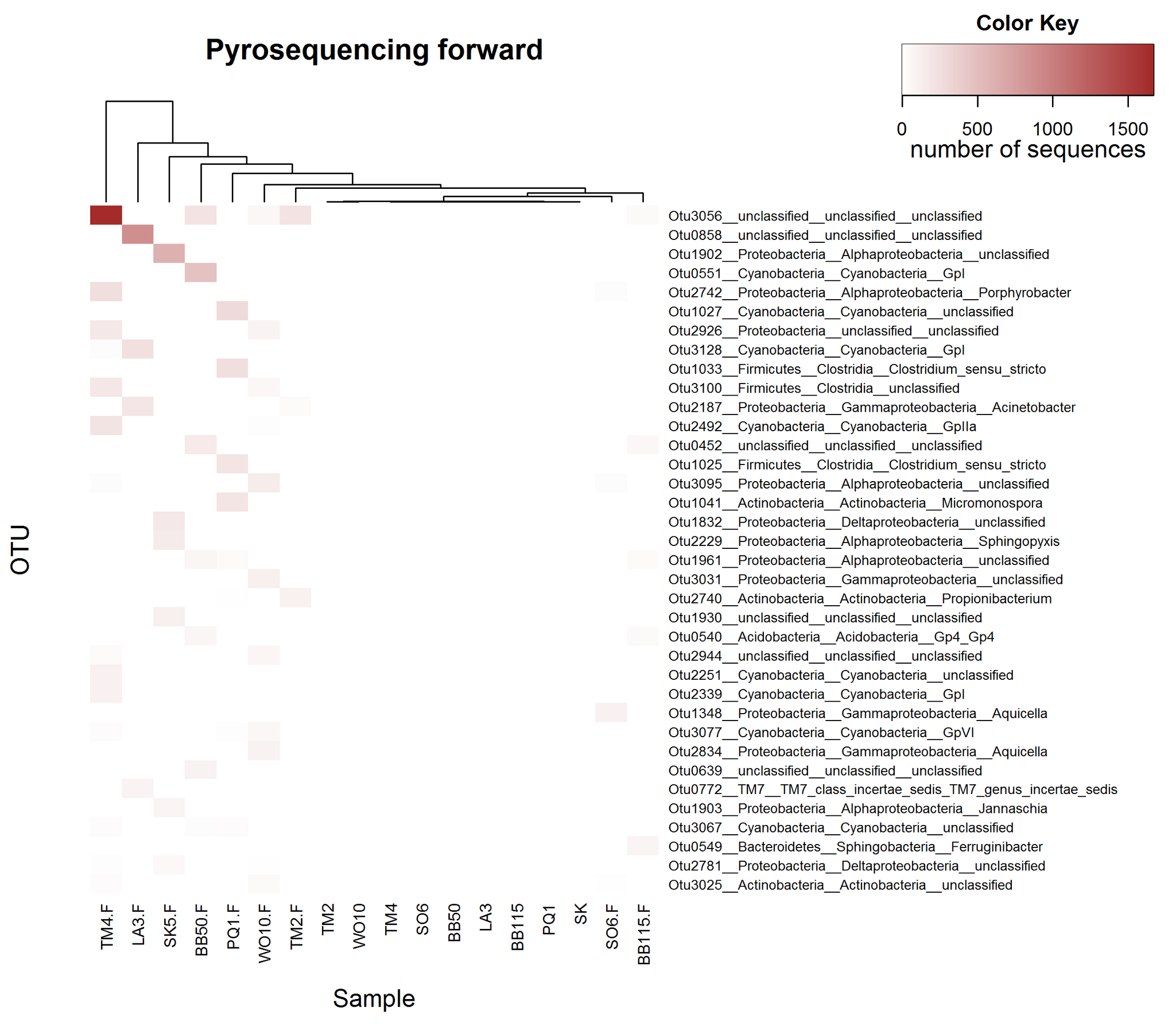

Supplement: Figure S3 — Heatmap showing the distribution of the most abundant OTUs based on forward pyrosequencing. These high abundant OTUs are represented by at least 80 sequences. Pyrosequenced samples have the suffix.F. (TIFF) [file pone.0097564.s003.tiff]

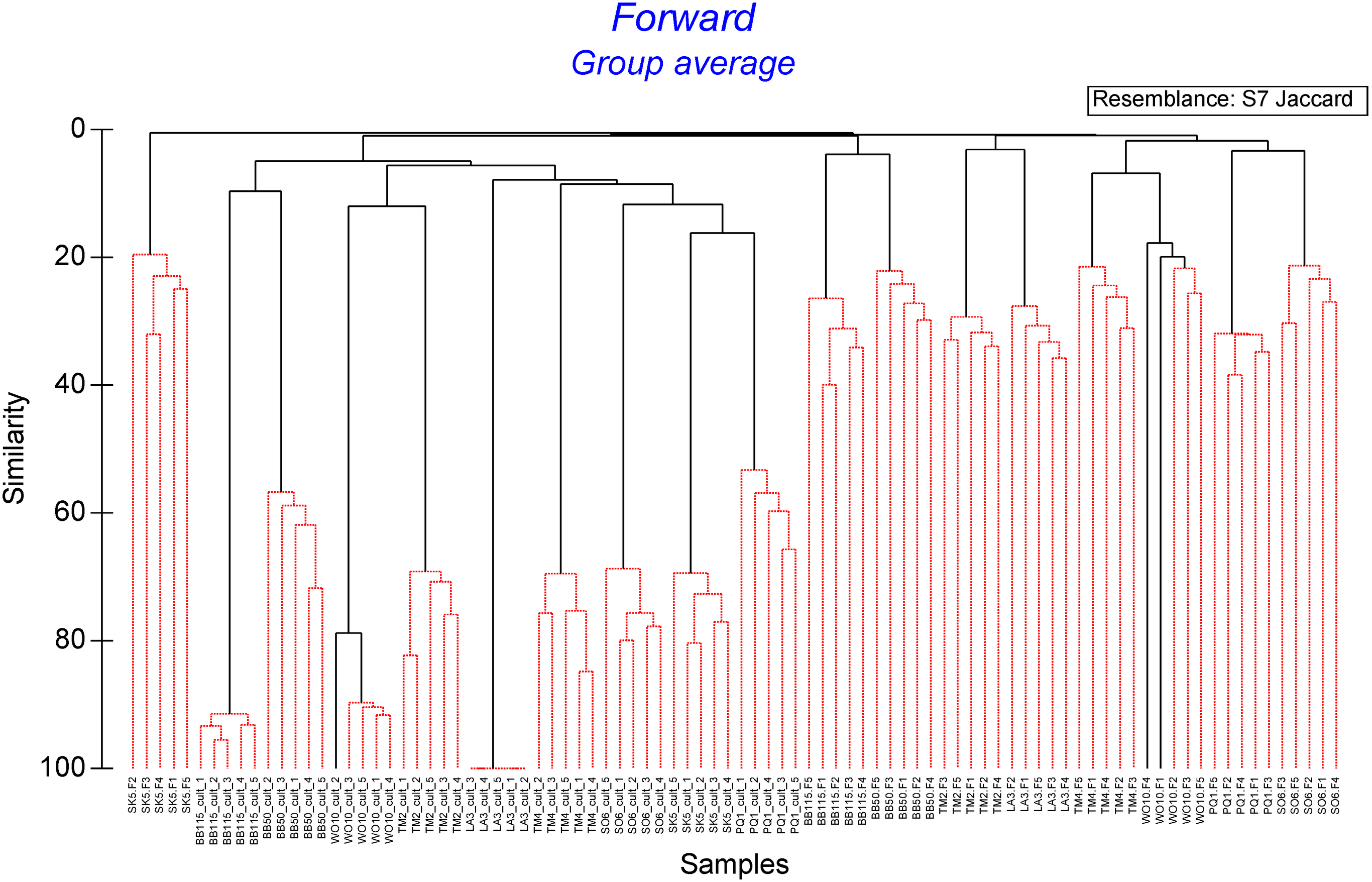

Supplement: Figure S4 — SIMPROF showing the clustering of the forward dataset. Each sample was subsampled 5 times with replacement to the lowest number of sequences (119 in cultured sample LA3). Full (black) lines are significant, dashed (red) lines are not. (TIF) [file pone.0097564.s004.tif]

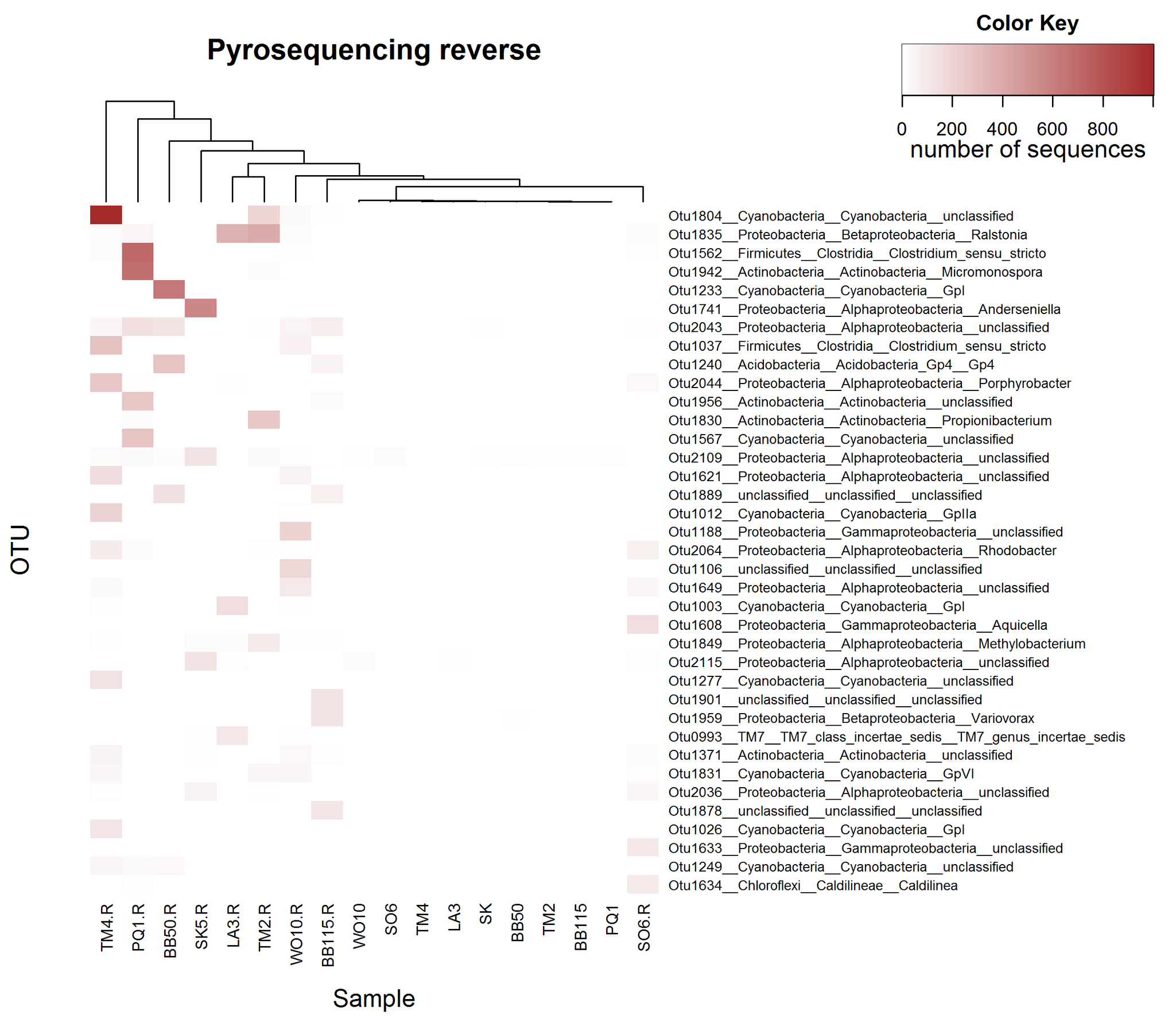

Supplement: Figure S5 — Heatmap showing the distribution of the most abundant OTUs based on reverse pyrosequencing. These high abundant OTUs are represented by at least 100 sequences. Pyrosequenced samples have the suffix.R. (TIFF) [file pone.0097564.s005.tiff]

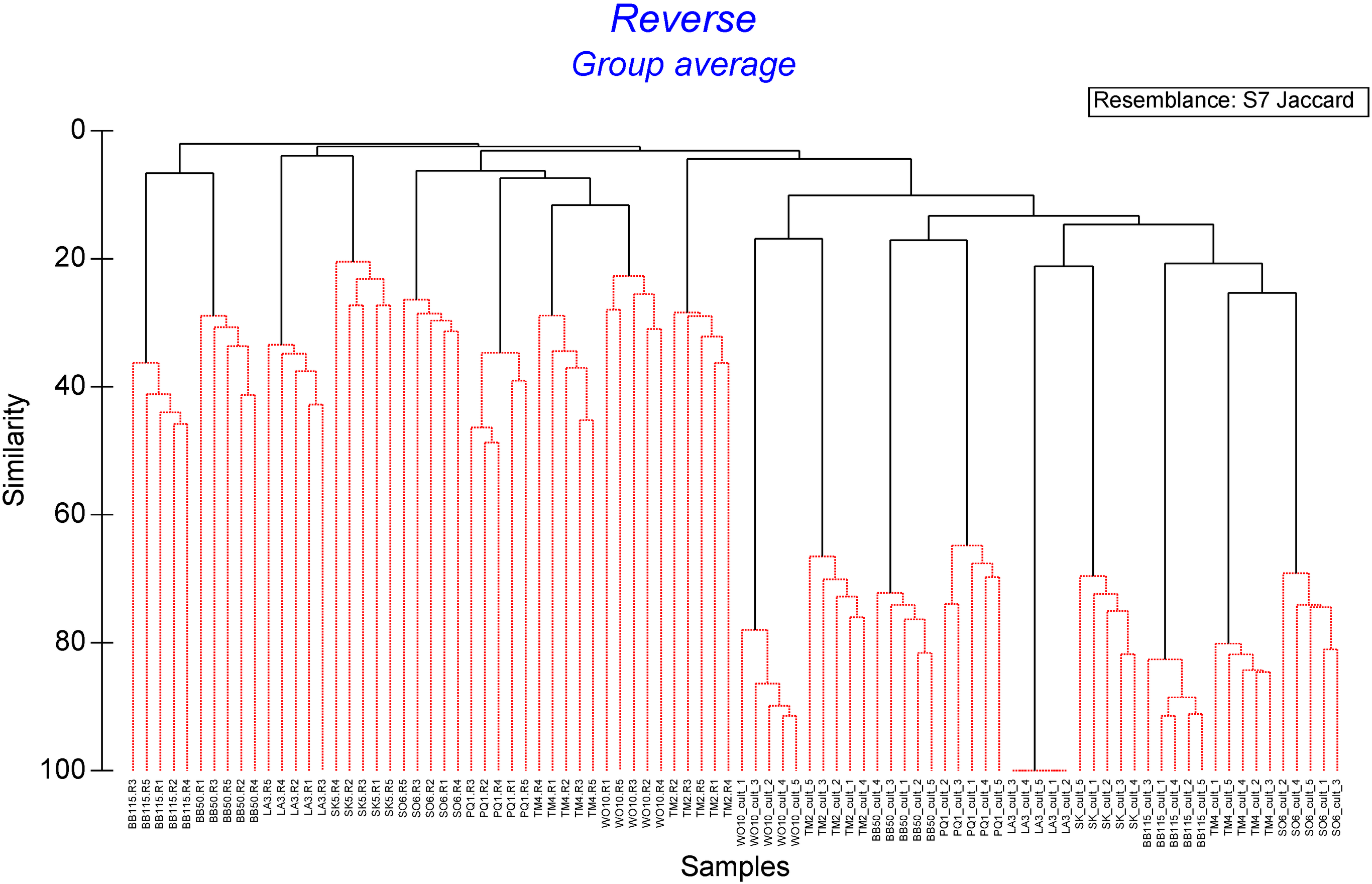

Supplement: Figure S6 — SIMPROF showing the clustering of the reverse dataset. Each sample was subsampled 5 times with replacement to the lowest number of sequences (116 in cultured sample LA3). Full (black) lines are significant, dashed (red) lines are not. (TIF) [file pone.0097564.s006.tif]

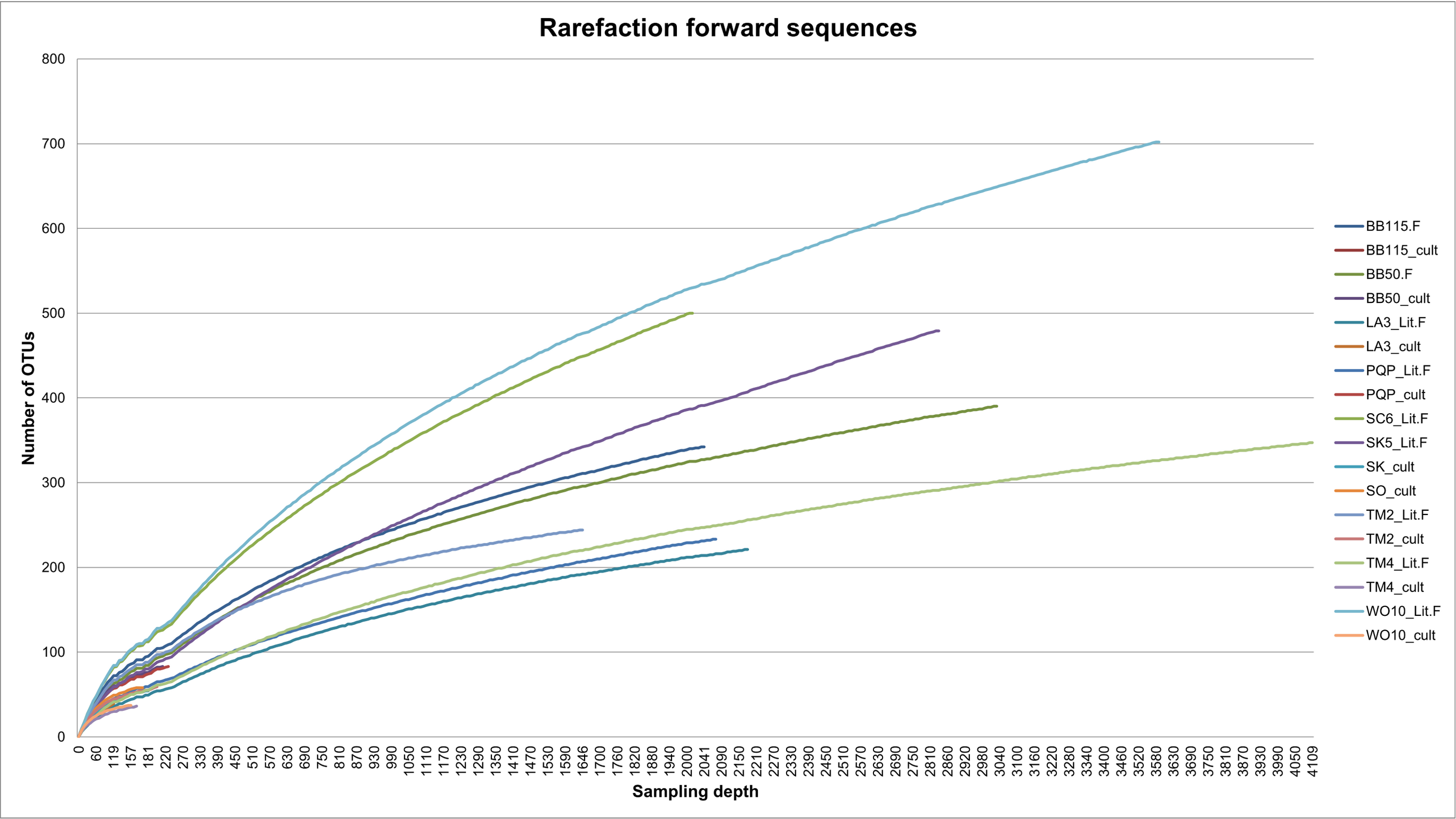

Supplement: Figure S7 — Rarefaction of the forward sequenced samples. (TIF) [file pone.0097564.s007.tif]

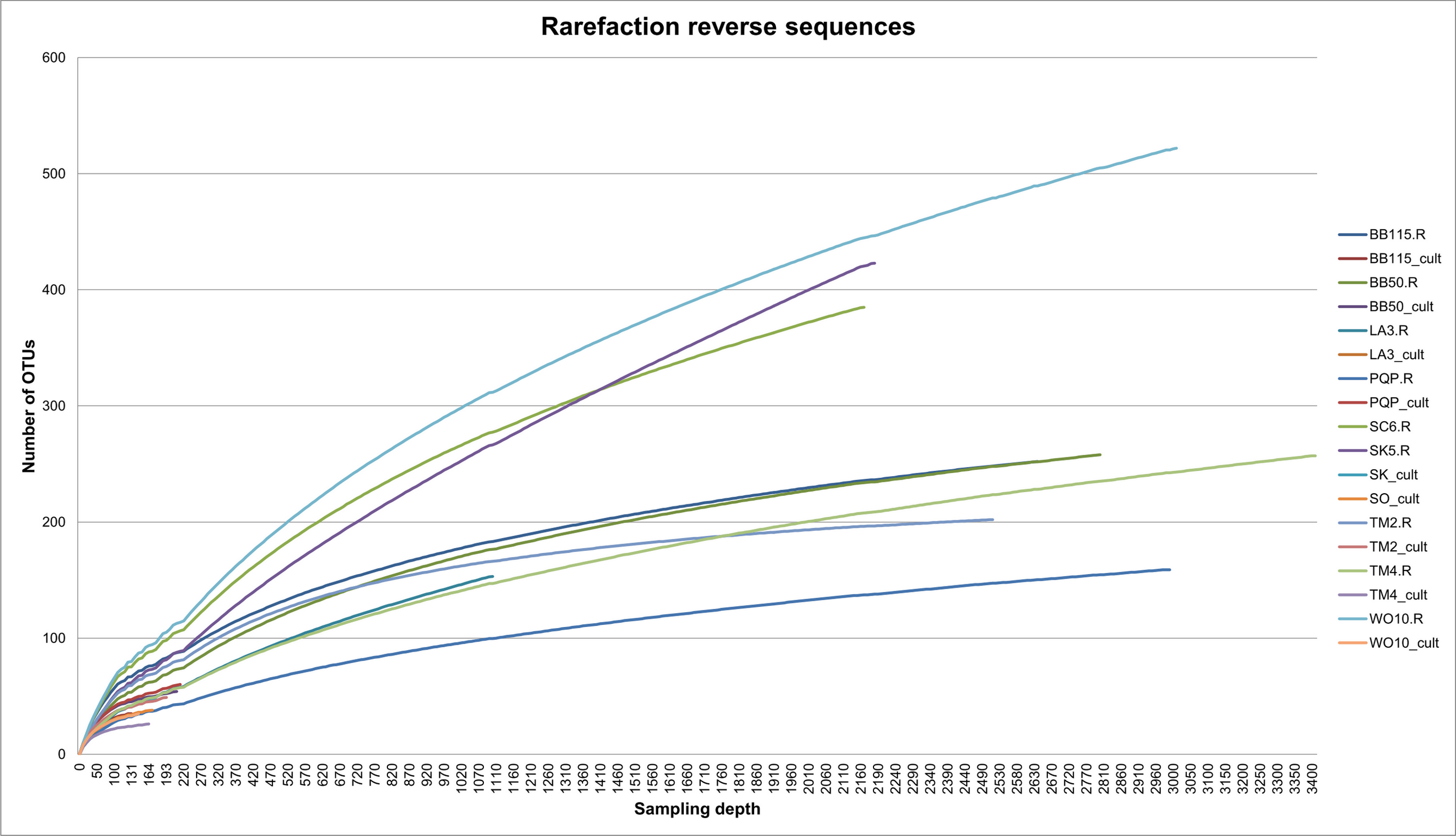

Supplement: Figure S8 — Rarefaction of the reverse sequenced samples. (TIF) [file pone.0097564.s008.tif]
